# China’s anti-malaria development assistance for health to sub-Saharan Africa and its influencing factors: A panel analysis

**DOI:** 10.7189/jogh.14.04250

**Published:** 2024-12-20

**Authors:** Junyi Shi, Yikai Feng, Minmin Wang, Mailikezhati Maimaitiming, Yinzi Jin, Minghui Ren, Zhijie Zheng

**Affiliations:** 1 Department of Global Health, School of Public Health, Peking University, Beijing, China; 2 Institute of Global Health, Peking University, Beijing, China; 3 China Centre for Health Development Studies, Peking University, Beijing, China

## Abstract

**Background:**

Malaria is a serious global health concern, with sub-Saharan Africa remaining the most burdened region. Despite China’s emphasis on overseas anti-malaria development assistance for health, its contributions to malaria prevention and control in sub-Saharan Africa are understudied. In this study, we examined the current situation of China’s anti-malaria development assistance for health and its influencing factors to support strategy development for optimising the practice in the future.

**Methods:**

We estimated the funding amounts based on AidData’s Global Chinese Development Finance Data set, version 3.0. We employed panel regression to explore the influencing factors, with the project funding amounts as the dependent variables and the indicators of the recipient countries’ level of health and health system, economic and political characteristics, and relationship with China as the explanatory variables. We conducted semi-structured interviews to acquire insights and elaboration from relevant agencies.

**Results:**

Between 2000–21, China provided anti-malaria assistance to 36 sub-Saharan African countries with 279 projects and an estimated sum of USD 319.81 million. The main projects were infrastructure construction (USD 181.22 million) and medicine donation (USD 122.14 million). The allocation of the total amount was correlated with the universal health coverage service coverage indexes (coefficient = –0.200; 95% confidence interval (CI) = –0.305, –0.095, *P* < 0.001) and the accountability rating in the public sector (coefficient = 0.121; 95% CI = 0.030, 0.212, *P* = 0.009). Such findings suggested China’s assistance tended to be directed to those countries with poorer capacity in the primary health system and better capacity for governance.

**Conclusions:**

China has made considerable contributions to malaria prevention and control in this region, yet the findings also pointed out the lack of systematic emphasis on capacity building. To strengthen the independent capacity and resilience of the recipient countries, it would be more effective for China and other donors to expand the types of anti-malaria projects and pay more attention to sustainable health system strengthening, including workforce training and primary care facility empowerment. These measures are only possible with increased funding.

Malaria is a major public health challenge worldwide, especially in sub-Saharan Africa (SSA), where the malaria disease burden remains the highest globally. In 2022, the SSA region accounted for 95% of total malaria cases; the deaths in this region reached 580 000, and four SSA countries accounted for approximately half of the global mortality [1]. At present, malaria prevention and control in Africa is faced with structural challenges such as insufficient funds, weak health systems, and a grave shortage in the health workforce; these adverse factors are impeding the implementation of preventive and case management measures, the domestic capacity for research and development, and the responses to emerging challenges including drug resistance [2,3].

Against such a backdrop, greater financial, material, and intellectual investments are required to promote eliminating malaria. Development assistance for health (DAH) has always been an important source of in-kind and monetary support for malaria prevention and control. Since the 2010s, about 75% of the total fundings for malaria control and elimination in SSA has been sourced from global donors [1]; in 2021, the global anti-malaria DAH reached USD 2.4 billion, constituting 3.58% of the total DAH [4]. Yet, as the shortage of resources in the SSA has not been relieved, more capable actors are desperately needed to be mobilised and join the DAH enterprise.

After a 70-year struggle against malaria, in 2021 China was certified as malaria-free by the World Health Organization (WHO) [5,6], and the valuable lessons learned during this process are worth sharing with other malaria-burdened countries. Malaria prevention and control has been an important aspect of China’s DAH. As was reported, China funded a total of 1339 health-related projects between 2000–17. 75% of these projects were carried out in Africa, and 18% belonged to the category of malaria prevention and control [7]. However, no study has conducted detailed analyses on the scale, types, and other characteristics of China’s overseas anti-malaria projects, and therefore, it is impossible to measure the contribution of China and prompt next-step improvement and innovation. In this study, we aimed to comprehensively map the financial input and flow, the project types, and the factors influencing China’s anti-malaria DAH to SSA. The findings provide evidence to support the future decision-making of the relevant agencies.

## METHODS

### Variables and data sources

We acquired data on the amounts and project details of China’s DAH for malaria from AidData’s Global Chinese Development Finance Data set (version 3.0), which was developed by the College of William & Mary and records known projects (with developmental, commercial, or representational intent) supported by official financial and in-kind commitments (or pledges) from China between 2000–21 [8]. In this study, we identified the content, amount, and temporal and spatial distribution of DAH projects in the descriptive analysis, and the amount is used as the dependent variable in the influencing factor analysis.

For the explanatory variables, we first reviewed existing literature on influencing factors of DAH, and we found three categories of variables to be often examined in such analyses. Variables in the first category reflect the health status and the health system of the recipient countries, including life expectancy, all-cause disease burden and of certain diseases, intervention and treatment coverage of certain diseases, and health expenditure [9–11]. Variables in the second category reflect the economic and political characteristics of the recipient countries, including gross domestic product or gross national income per capita, population, natural resource rent (reflecting the importance of natural resources for the national economy), and indicators extracted from the World Governance Indicators [9–11]. Variables in the third category reflect the relationship between the recipient countries and the donor countries, including market openness rate and other indicators for trade relations, and voting consistency in the United Nations General Assembly [9–14]. We examined the factors of these three categories to influence donors’ allocation of development assistance to different extents.

Considering the pertinency and the availability of the corresponding data, we included the variables in this study (**Table 1**). We considered additional indicators for diplomatic relationships, including Chinese workers in the recipient countries and a number of bi-directional diplomatic visits. For the variables reflecting the malaria disease burden, we included seven more indicators for sensitivity analysis, and it is the same for the variables reflecting health expenditure and universal health coverage (UHC).

**Table 1:** Explanation variables of the influencing factor analysis

Category and variable name	Time	Source
Health and health system		
*Malaria DALY in number*	2000–19	GBD 2019, IHME
*Malaria death in number*	2000–19	GBD 2019, IHME
*Malaria incidence in number*	2000–19	GBD 2019, IHME
*Malaria prevalence in number*	2000–19	GBD 2019, IHME
*Malaria DALY rate (per 100 000 population)*	2000–19	GBD 2019, IHME
*Malaria death rate (per 100 000 population)*	2000–19	GBD 2019, IHME
*Malaria incidence rate (per 100 000 population)*	2000–19	GBD 2019, IHME
*Malaria prevalence rate (per 100 000 population)*	2000–19	GBD 2019, IHME
*GGHE-D%CHE*	2000–21	WHO Global Health Expenditure Database
*Infectious %GGHE-D*	2000–21	WHO Global Health Expenditure Database
*UHC-SCI (0 = low, 100 = high)*	2000, 2005, 2017, 2019, 2021	WHO Global Health Observatory UHC-SCI (3.8.1)
*UHC sub-index for infectious diseases*	2000, 2005, 2017, 2019, 2021	WHO Global Health Observatory UHC-SCI (3.8.1)
Economic and political characteristics		
*GNI per capita*	2000–21	World Bank, World Development Indicators data set
*Natural resource rents as % of GDP*	2000–21	World Bank, World Development Indicators data set
*TAC in the public sector rating (1 = low, 6 = high)*	2000–21	World Bank, World Development Indicators data set
*PSA estimate (–2.5 = low, 2.5 = high)*	2000–21	World Bank, World Development Indicators data set
Relationship with China		
*China’s exports*	2000–21	China Africa Research Initiative at Johns Hopkins University’s School of Advanced International Studies
*China’s imports*	2000–21	China Africa Research Initiative at Johns Hopkins University’s School of Advanced International Studies
*China’s investment*	2003–21	China Africa Research Initiative at Johns Hopkins University’s School of Advanced International Studies
*Number of Chinese workers*	2009–21	China Africa Research Initiative at Johns Hopkins University’s School of Advanced International Studies
*Voting consistency in the UNGA*	2000–21	Harvard Dataverse, United Nations General Assembly Voting Data
*Bi-directional diplomatic visits*	2000–21	China International Development Cooperation Agency

DALY – disability-adjusted life years, GBD – Global Burden of Disease Study, GGHE-D%CHE – domestic general government health expenditure as a percentage of current health expenditure, GNI – gross national income, IHME – Institute for Health Metrics and Evaluation, PSA – political stability and absence of violence/terrorism, TAC – transparency, accountability, and corruption, UHC-SCI – universal health coverage service coverage index, UNGA – United Nations General Assembly, WHO – World Health Organization, %GGHE-D – percentage of domestic general government health expenditure

### DAH project categorising and amount estimating

For descriptive analysis, we extracted China’s DAH projects for malaria in SSA from AidData’s data set. We first categorised them according to their contents. The framework we used in the categorising process combined the WHO Guidelines for Malaria, version 6.1 [15] and the building blocks framework of the health system [16]. The former provides intervention guidance by categories, including prevention, case management, and surveillance, while the latter provides a general classification of the health system’s fundamental factors (**Table 2**).

**Table 2:** The integrated categorising framework for anti-malaria DAH projects

Building blocks and categories	Secondary category
Leadership and governance	
*Policy formulation*	NA
*Social mobilisation*	NA
Health information system	
*Surveillance*	NA
Resource	
*Financing*	NA
*Facility*	NA
Health workforce	
*Training for human resources for health*	NA
Goods and services delivery	
*Prevention*	Vector control, chemoprevention, etc.
*Case management*	Diagnosis, treatment, etc.

NA – not available

Of the 281 anti-malaria projects in SSA recorded in the AidData data set, 54.45% of the allocated amount was missing. To fill in the missing values for the allocated amounts, we assumed that the projects in the same category resemble one another in terms of the allocated amount, and thus, we calculated the mean value of the allocated amounts in each type, and we used it to replace the missing values. We described the estimated allocated amounts, project counts, and spatial and temporal distribution of the projects. The monetary unit is 2021 constant USD.

### Panel analysis

In analysing factors influencing China’s DAH for malaria in SSA, we used the amount of DAH as the dependent variable. In panel regression, there are three possible methods, namely, the common constant method (CC), also called the pooled ordinary least squares method, the fixed effects method (FE), and the random effects method (RE). The CC method assumes that no difference lies among the data matrices of the cross-sectional dimensions and estimates one common intercept for all groups (in this case, countries). The FE method assumes differences among the data matrices and allows for a different intercept for each country. The RE method assumes the intercepts for each country are random parameters, meaning the unobserved country-specific effects are not correlated with the explanatory variables [17].

We first conducted the Breusch-Pagan test to decide between the two variable intercept models and the CC model, and we used the Hausman specification test to decide between the FE method against the RE method. Seeing assistance decision-making was based on lagging indicators, in this study, the explanatory variables were two to six-year-lagged; we chose the best model with the largest adjusted *R^2^*, which represented the proportion of the variance explained by the independent variables in each model. We decided on such a lag period based on the frequency of national health-related surveys in SSA and the self-reported experience of officers working in the relevant agencies.

Since the indicators included in the analysis came in different units and orders of magnitude, we needed to standardise the variables needed to acquire comparable coefficients. As the data appeared to scatter at a wide range and cluster at some points, we chose the Z-score method over the other methods to clarify and amplify the data distribution. We set α to be 0.10, meaning statistical significance existed when the *P*-value was <0.10. We used Stata, version 17.0 (StataCorp LLC, College Station, Texas, USA) to organise the data and conduct analyses.

### Semi-structured interviews

To better explain the results and gain insights on this topic, we conducted semi-structured interviews with eight experts involved in the decision-making or the implementation of China’s anti-malaria DAH. The experts remain anonymous in this article, and their affiliations are listed in Table S1 in the **Online Supplementary Document**. The questions covered the detailed management procedure of DAH projects in China, their explanations of the results of the statistical analyses in this study, and their opinions on the advantages, disadvantages and insufficiencies of China’s anti-malaria DAH.

## RESULTS

### Contents of China’s anti-malaria DAH

Between 2000–21, China provided support for three types of building blocks, and they are resources, health workforce, and goods and services. For resources, the infrastructure construction projects included constructing 31 anti-malaria centres and one laboratory and renovating one anti-malaria centre and one laboratory. For the health workforce, the training programs were conducted by sending Chinese experts to the recipient countries or funding staff from the recipient countries to receive training in China. For goods and services, the projects covered vector control, chemoprevention, diagnosis, and treatment, including indoor residual spraying project, insecticide-treated nets donation, mass drug administration, diagnosis supply donation, and medicine donation. In addition, the Chinese medical teams provided direct diagnosis and treatment services to the local patients, but only seven such projects were recorded explicitly as relevant in the data set; the other Chinese medical teams may undertake such work as well, but it is unmeasurable based on the existing information. The total project count of China’s DAH for malaria in SSA reached 279, and the estimated amount reached USD 319.81 million. The largest proportion of funding (56.07%) has been put into infrastructure construction. Medicine donations were the second most invested type of DAH, accounting for 38.96% of the total funding, while its project counts appeared to be far beyond the other types (**Table 3**).

**Table 3:** Contents of China’s anti-malaria DAH to SSA, 2000–21

Building block and category	Secondary category	Content	Estimated amount in USD millions	Projects (n)
Resource				
*Facility*	NA	Infrastructure construction	181.22	34
Health workforce				
*Training for HRH*	NA	Training for technicians, physicians, and officials	1.00	17
Goods and service				
*Prevention*	Vector control	IRS conduction	2.40	1
*Prevention*	Chemoprevention	MDA conduction	1.17	2
*Case management*	Diagnosis	Supply donation	8.73	16
*Case management*	Treatment	Medicine donation	122.14	195
*Case management*	Treatment	Direct service provision by CMT	1.24	7
Total			319.81	279

CMT – Chinese medical teams, HRH – human resource for health, IRS – indoor residual spraying, ITN – insecticide-treated nets, MDA – mass drug administration, NA – not applicable

### Temporal and spatial distribution of China’s DAH

**Figure 1:**
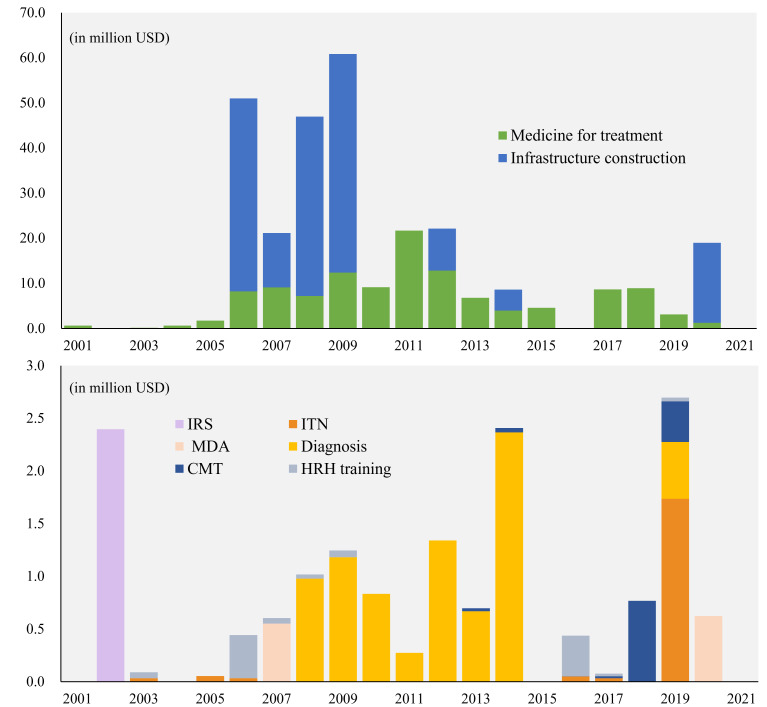
Project types and estimated amounts of China’s anti-malaria DAH in SSA. DAH – development assistance for health, SSA – sub-Saharan Africa.

The funding fluctuated greatly during the studied period, and most funding was invested between 2006–09. At the China-Africa Cooperation Forum in 2006, China promised to build 30 anti-malaria centres in Africa, and these projects appeared intensively during this period. Medicine donation was the longest-running project type and appeared each year. The funding for such projects first increased steadily and peaked in 2011, reaching an estimated USD 21.68 million, and then dropped in an undulatory way. Most of the diagnosis supply donation projects appeared between 2008–14 and were often combined with medicine donations. However, the funding was unstable, and the volume was limited comparingly (**Figure 1**).

**Figure 2:**
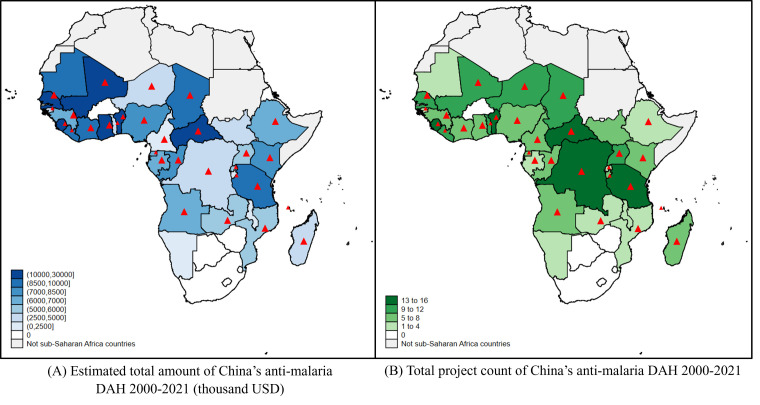
Distribution of recipient countries of China’s anti-malaria DAH and the estimated amounts and project counts. Red triangles mark the 30 recipient countries where anti-malaria centres were built. DAH – development assistance for health.

From the aspect of spatial distribution, 36 of the 46 SSA countries had received anti-malaria DAH from China. However, the estimated amount and project count in each recipient country did not always match. For instance, in the Democratic Republic of the Congo, the estimated allocated amount (USD 5.12 million) ranked 30th, and the project count (n = 14) ranked fourth. The mismatch might result from long-term contracts between some countries and China. As recorded in the data set, China would provide one batch of anti-malaria medicine annually for these countries. Thus, the aggregated project count was high, but the scale of funding for each batch was not very large comparingly. For other countries, there were no long-term contracts but infrastructure construction projects, resulting in low project counts but high estimated amounts (**Figure 2**).

**Figure 3:**
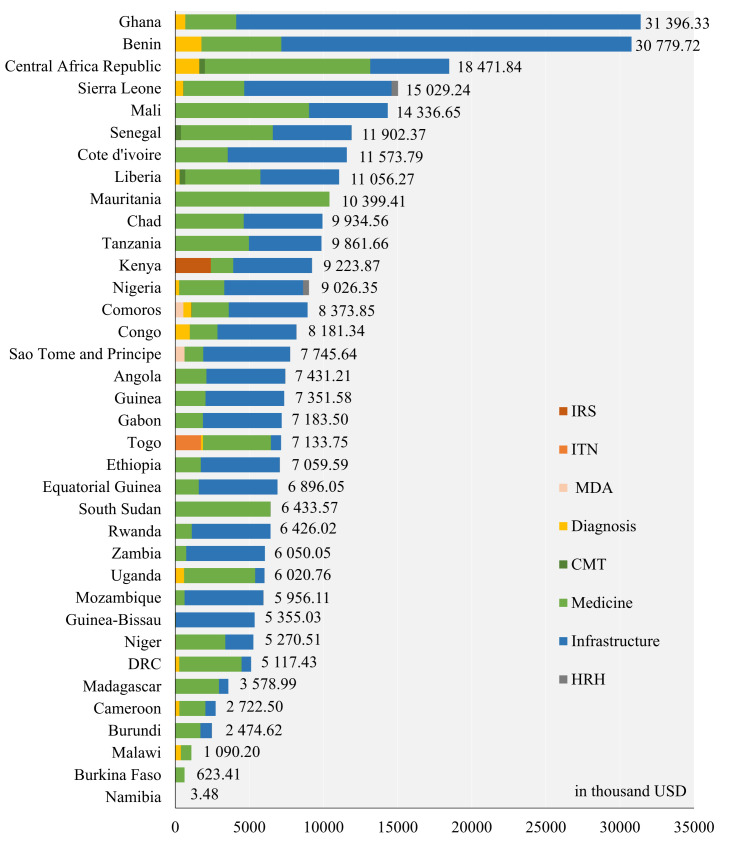
Project types and estimated amounts of China’s anti-malaria DAH by country. DAH – development assistance for health.

From the aspect of recipient countries, Ghana received the highest DAH fundings of an estimated USD 31.40 million and Benin the second (USD 30.78 million), while Burkina Faso and Namibia received the lowest fundings (USD 0.62 million and USD 3.50 thousand). Only one project for Namibia was recorded in the data set, which was a training program after the flood in 2009. In 19 countries, infrastructure construction funding took up over half of the total funding. All the countries had received medicine donations except Namibia and Guinea-Bissau. Indoor residual spraying projects were conducted only in Kenya, and mass drug administration projects were conducted only in Comoros and Sao Tome and Principe. The mass drug administration in Comoros that started in 2007 contributed greatly to controlling the local malaria prevalence. By 2014 the case number had decreased by 98% from 100 000 and achieved zero death (**Figure 3**).

### Influencing factors of China’s anti-malaria DAH to SSA

For the factors influencing the total amount allocation, we first conducted the Breusch-Pagan test, and the CC model was chosen for panel regression. The five-year lagged models appeared to be larger in *R^2^*, which suggested a better goodness-of-fit, and thus, we took it as the basis for result interpretation. For the variables indicating the health status of the recipient countries, all the rates of malaria disease burden are positively correlated with the allocated amount, and the universal health coverage service coverage index (UHC-SCI) negatively. For the variables indicating the economics and politics of the recipient countries, the transparency, accountability, and corruption (TAC) rating is positively correlated with the amount and the gross national income per capita negatively. For the variables indicating the recipient countries’ relationship with China, China’s exports, investment and number of Chinese workers negatively correlate to the amount (**Table 4**). The complete results of two to six-year lagged panel regression of all the 23 variables are shown in Table S2 in the **Online Supplementary Document**. We conducted the robustness analyses with the missing values dropped and the results are in accordance with the results here in general (Table S4 in the **Online Supplementary Document**).

**Table 4:** Univariable panel analysis of influencing factors of the total amount allocation

	Three-year lagged		Four-year lagged			Five-year lagged	
**Categories and variables**	**Coefficient (95% CI)**	***P*-value**	**Coefficient (95% CI)**	***P*-value**		**Coefficient (95% CI)**	***P*-value**
Health and health system							
*Death rate*	0.080 (0.000, 0.159)*	0.050*	0.084 (0.001, 0.167)*		0.047*	0.090 (0.003, 0.176)*†	0.043*
*UHC sub-index on infectious diseases*	–0.112 (–0.197, –0.027)*	0.009*	–0.149 (–0.240, –0.058)*		0.001*	–0.172 (–0.271, –0.072)*†	0.001*
Economics and politics							
*TAC rating*	0.079 (–0.003, 0.161)*	0.059*	0.092 (0.005, 0.178)*		0.038*	0.095 (0.003, 0.187)*†	0.043*
*GNI per capita*	–0.048 (–0.127, 0.030)	0.226	–0.074 (–0.155, 0.008)*		0.078*	–0.091 (–0.177, –0.006)*†	0.037*
Relationship with China							
*China’s exports*	–0.051 (–0.142, 0.040)	0.270	–0.078 (–0.176, 0.019)		0.116	–0.100 (–0.205, 0.006)*†	0.064*
*China’s investment*	–0.101 (–0.200, –0.001)*	0.048*	–0.118 (–0.237, 0.001)*		0.053*	–0.145 (–0.285, –0.005)*†	0.042*
*Number of Chinese workers*	–0.080 (–0.157, –0.004)*	0.040*	–0.087 (–0.168, –0.006)*†		0.036*	–0.089 (–0.175, –0.003)*	0.042*

CI – confidence interval, GNI – gross national income, TAC – transparency, accountability and corruption, UHC – universal health coverage

*Statistically significant.

†Model with the largest adjusted *R^2^*.

We subsequently conducted multivariable panel analyses with the variables listed above. For similar variables (i.e. the eight indicators for malaria disease burden), we conducted sensitivity analyses, and we included one variable in the final model based on the adjusted *R^2^*. Only UHC-SCIs and TAC ratings showed statistical significance. Such results suggest that the capacity for basic health service provision and government administration are key factors influencing the allocation of the total amounts of China’s anti-malaria DAH (**Table 5**).

**Table 5:** Multivariable panel analysis of influencing factors of the total amount allocation

	Three-year lagged		Four-year lagged		Five-year lagged	
**Categories and variables**	**Coefficient (95% CI)**	***P*-value**	**Coefficient (95% CI)**	***P*-value**	**Coefficient (95% CI)**	***P*-value**
Health and health system						
*Death rate*	0.024 (–0.072, 0.121)	0.624	0.023 (–0.065, 0.113)	0.600	0.032 (–0.060, 0.125)	0.494
*UHC sub-index on infectious diseases*	–0.261 (–0.388, –0.133)*	0.000*	–0.200 (–0.305, –0.095)*	0.000*	–0.210 (–0.326, –0.094)*	0.000*
Economics and politics						
*TAC rating*	0.129 (0.028, 0.231)*	0.012*	0.121 (0.030, 0.212)*	0.009*	0.128 (0.031, 0.223)*	0.009*
*GNI per capita*	–0.044 (–0.140, 0.051)	0.364	–0.025 (–0.112, 0.061)	0.560	–0.040 (–0.131, 0.051)	0.384
Relationship with China						
*China’s exports*	–0.055(–0.190, 0.079)	0.420	–0.041 (–0.156, 0.073)	0.479	–0.059 (–0.184, 0.067)	0.360
*China’s investment*	–0.052 (–0.227, 0.121)	0.552	–0.015 (–0.110, 0.079)	0.752	–0.003 (–0.103, 0.095)	0.700
*Number of Chinese workers*	0.004 (–0100, 0.109)	0.940	–0.007 (–0.095, 0.079)	0.861	–0.017 (–0.112, 0.078)	0.894

CI – confidence interval, GNI – gross national income, TAC – transparency, accountability and corruption, UHC – universal health coverage

*Statistically significant.

Considering medicine donation is the type of DAH that lasted for the longest time and covered most recipient countries, in the following section we present its influencing factors exclusively. Based on the result of the Breusch-Pagan test, we chose the CC model for panel regression. We used the five-year lagged models as the basis for result interpretation. For the variables indicating the health status of the recipient countries, no indicator of malaria burden showed statistical significance, while the domestic general government health expenditure as a percentage of current health expenditure and the UHC-SCI both showed a negative correlation. The rest of the results were similar to the results above, except that the TAC rating showed no statistical significance (**Table 6**). The complete results of two to six-year lagged panel regression of all the 23 variables are shown in Table S3 in the **Online Supplementary Document**. We conducted the robustness analyses with the missing values dropped, and the results are in accordance with the results here in general (Table S5 in the **Online Supplementary Document**).

**Table 6:** Univariable panel analysis of influencing factors of the allocation of medicine

	Three-year lagged		Four-year lagged		Five-year lagged	
**Categories and variables**	**Coefficient (95% CI)**	***P*-value**	**Coefficient (95% CI)**	***P*-value**	**Coefficient (95% CI)**	***P*-value**
Health and health system						
*GGHE-D%CHE*	–0.068 (–0.149, 0.013)	0.101	–0.076 (–0.161, 0.009)*†	0.082*	–0.063 (–0.154, 0.027)	0.173
*UHC sub-index on infectious diseases*	–0.124 (–0.209, –0.039)*	0.004*	–0.133 (–0.224, –0.042)*	0.004*	–0.165 (–0.264, –0.066)*†	0.001*
Economics and politics						
*GNI per capita*	–0.061 (–0.139, 0.017)	0.126	–0.079 (–0.161, 0.002)*	0.056*	–0.096 (–0.181, –0.010)*†	0.027*
Relationship with China						
*China’s exports*	–0.049 (–0.141, 0.041)	0.284	–0.079 (–0.177, 0.018)	0.110	–0.103 (–0.208, 0.001)*†	0.054*
*China’s investment*	–0.095 (–0.195, 0.003)*	0.059*	–0.078 (–0.197, 0.040)	0.197	–0.148 (–0.287, –0.008)*†	0.037*
*Number of Chinese workers*	–0.070 (–0.147, 0.005)*	0.071*	–0.088 (–0.169, –0.007)*	0.033*	–0.094 (–0.180, –0.009)*†	0.030*

CI – confidence interval, GGHE-D%CHE – domestic general government health expenditure as a percentage of current health expenditure, GNI – gross national income, UHC – universal health coverage.

*Statistically significant.

†Model with the largest adjusted *R^2^*.

We subsequently conducted multivariable panel analyses with the variables listed above. For similar variables, we conducted sensitivity analyses, and one variable was included in the final model based on the adjusted *R^2^*. Only UHC-SCIs showed statistical significance. It can be inferred that the capacity for basic health service provision of the recipient countries would influence the allocation of China’s anti-malaria DAH primarily, accorded with the global appeal for health equity. When the administrative progress of a DAH project is complex and demands inter-government coordination, the transparency and accountability of the recipient government would become an important factor for consideration (**Table 7**).

**Table 7:** Multivariable panel analysis of influencing factors of the allocation of medicine

	Three-year lagged		Four-year lagged		Five-year lagged	
**Categories and variables**	**Coefficient (95% CI)**	***P*-value**	**Coefficient (95% CI)**	***P*-value**	**Coefficient (95% CI)**	***P*-value**
Health and health system						
GGHE -D%CHE	–0.021 (–0.126, 0.083)	0.684	–0.043 (–0.137, 0.049)	0.359	–0.021 (–0.119, 0.078)	0.681
UHC sub-index on infectious diseases	–0.160 (–0.283, –0.037)*	0.011*	–0.098 (–0.199, 0.002)*	0.056*	–0.114 (–0.227, –0.003)*	0.044*
Economics and politics						
GNI per capita	–0.054 (–0.154, 0.044)	0.281	–0.032 (–0.123, 0.058)	0.479	–0.052 (–0.147, 0.042)	0.274
China’s exports	–0.030 (–0.163, 0.101)	0.648	–0.033 (–0.147, 0.080)	0.565	–0.031 (–0.155, 0.092)	0.619
Relationship with China						
China’s investment	–0.108 (–0.282, 0.066)	0.223	0.002 (–0.134, 0.138)	0.975	–0.067 (–0.226, 0.090)	0.581
Number of Chinese workers	0.009 (–0.094, 0.114)	0.734	–0.037(–0.134, 0.058)	0.440	–0.031 (–0.131, 0.069)	0.542

CI – confidence interval, GGHE-D%CHE – general government health expenditure as a percentage of current health expenditure, GNI – gross national income, UHC – universal health coverage.

*Statistically significant.

## DISCUSSION

### On the estimated funding amounts and the project contents

There were two previous articles reporting the estimated amount of China’s DAH based on different versions of AidData’s data set. Shajalal categorised the DAH projects with the framework of the Creditor Reporting System of the Organization for Economic Co-operation and Development and estimated the total amount between 2000–13 to be USD 2.16 billion, and about USD 180 million was allocated for malaria prevention and control, yet the method for missing value filling was not reported [18]. McDade categorised the projects between 2000–17 with the frameworks of the Institute for Health Metrics and Evaluation and the Creditor Reporting System and filled in the missing values using the median value in each category of the projects [19]. By the former method, the total amount was USD 3.68 billion, while by the latter method, the total amount was USD 4.25 billion, with USD 113 million allocated for malaria prevention and control. Overall, it could be inferred that China’s funding for malaria prevention and control took up within 10% of the total DAH.

In this study, the total amount of China’s anti-malaria DAH to SSA between 2000–21 was estimated to reach USD 319.81 million, and the method for filling the missing values was to calculate the mean funding value of each type of project. Considering the comparingly high proportion of the missing values, such a method would surely introduce bias, and may over-estimate the summed amounts. Yet it is noteworthy that, on the one hand, as was recorded in the data set, the funding for each project appeared to be highly irregular and varied at a wide range. Therefore, neither mean value nor median value could be reckoned as fully representative. On the other hand, there remained considerable leave-outs of existing projects due to insufficient information disclosure from the relevant agencies, especially concerning projects of larger funding volumes. Nevertheless, the results of this study should be considered in combination with the existing literature.

From the perspective of project types, infrastructure construction was undoubtedly the most important approach for China to deliver anti-malaria DAH to SSA, while medicine donation is the only long-term approach to China’s anti-malaria DAH. Few other mature mechanisms have been identified in the other categories based on the data set, especially in vector control, surveillance, workforce establishment, and other possible means for strengthening the health system. However, a multi-faceted approach has always been the prerequisite for successful intervention projects. Taking the Fast Elimination of Malaria by Source Eradication project in Coromos for example, this mass drug administration project reduced the incidence rate on Moheli, Anjouan, and Grand Comoro islands by over 97% between 2007–14 [20,21]. Although it was documented and reported as a chemoprevention project, the implementation process also included sector-wide and cross-sector motivation, long-term training for primary health care workers and community volunteers, and support for mobile blood test stations. The success of the Fast Elimination of Malaria by Source Eradication project indicated that, in order to increase the effectiveness of DAH and accelerate malaria control and elimination in SSA, future projects should be designed as comprehensive and integrated.

### On the influencing factors of China’s anti-malaria DAH to SSA

During the studied period, in China’s official development assistance management system, there are two approaches for a DAH project to be established. In the first approach, a DAH project is requested by a recipient country. The request would be submitted first to the Chinese embassy and subsequently to the managing and coordinating agencies, including the Ministry of Foreign Affairs, Finance, and Commerce (since 2018, the duty has been transferred to China International Development Cooperation Agency) and the Ministry of Health (in 2013, Ministry of Health was re-organised and became National Health Commission). The relevant agencies would evaluate feasibility and decide whether and how to implement the project [22]. Thus, the establishment and implementation of these projects were influenced by both the recipient countries’ intentions and China’s capacity. In the second approach, the national ministries and commissions would regularly set up funds for DAH projects and the health-related institutions would apply for it. After expert review, the managing and coordinating agencies would negotiate with the implementing institutions to confirm the design and the budget of the eligible projects. Thus, the establishment and implementation of these projects are more affected by the intention and capacity of the health-related institutions of China.

The establishment approach of the projects included in this study cannot be ascertained according to the given information; it is therefore assumed that the impetus of China’s anti-malaria DAH is a mixture of the intentions of both the recipient countries and China. Thus, based on the results of the influencing factor analyses, one cannot assert the decision-making criteria or the purpose of China. It is only possible to describe preliminarily the inclination of anti-malaria DAH allocation towards certain kinds of recipient countries.

In this study, as was shown by the multivariable panel analyses, for the recipients’ health and health system, the UHC-SCI and the sub-index on infectious diseases were the sole variables of statistical significance. For the economics and politics of the recipients, only the TAC rating in the public sector showed statistical significance, and for the relationship with China, none of the indicators included in this study showed statistical significance. In comparison, some existing articles reported that China’s DAH allocation was correlated with health-related factors. For example, hospital allocation in Africa was correlated with the local facility delivery rate and under-five mortality rate [11], and the anti-malaria centre establishment was correlated with the local malaria disability-adjusted life years [10]. The UHC-SCI is a newly used indicator, and the lower index represents the nation’s poorer capacity of service provision and lower service accessibility for the people; the inclination towards such recipients indicated the significance of the health system capacity during the process of project development and decision making, and thus it was in accord with the existing findings to some extent. As to the TAC rating, the positive correlation suggested practical consideration of the relevant agencies to guarantee the quality of the projects and the effective usage of the financial resources. Apart from this, the results of this study did not support the criticism that China provides DAH to SSA countries in exchange for geopolitical interests [23–25].

### On the improvement of anti-malaria DAH

Since the beginning of the 21st century, malaria prevention and control has been a focus of China-Africa cooperation. In 2000, when the Forum on China-Africa Cooperation mechanism was established, treatment for malaria was listed as one of the areas of cooperation [26]. At the Beijing Summit in 2006, the Johannesburg Summit in 2015, the Beijing Summit in 2018, and the Dakar Summit in 2021, China committed to further cooperation in this area to accelerate malaria elimination in Africa in support of the malaria 2030 goals [27,28]. So far, China’s anti-malaria DAH to SSA has exhibited desirable consistency with the local capacity for service provision. However, the predictability and continuity of DAH allocation were less than ideal, and the forms of DAH were rather limited and not yet in full accordance with China’s experience, strength, and potential.

China’s successful domestic elimination of malaria was based on supportive factors across all the building blocks of health system and further, including government leadership and strict supervision, trans-region and trans-department cooperation, stable financial input, enhancement of health workforce, prevention and control measures that were adjusted to local conditions, careful implementation of surveillance, mass community participation, and encouragement for scientific innovation [29–32]. That is to say, in order to enhance a country’s independent capacity for prevention and control of malaria and other infectious diseases, sector-wide health system strengthening approaches need to be further emphasised both domestically and on the donor side. This is a caution not only for China but also for all the donors and recipients: every link of the health system, from top to bottom, requires optimising to ensure and improve the quality and the outcome of service.

To improve the quality and effectiveness of anti-malaria DAH, comprehensive and systematic strategies must first be developed. On the one hand, donors should continue and stabilise their financial contributions to supply procurement and delivery in support of routine services and campaigns [33,34]. On the other hand, it is of great importance to emphasise capacity building in the recipient countries, with experience sharing and workforce training serving as the basis for the recipients to enhance their own anti-malarial health system. Besides this, the local or cross-border health information system forms the basis for evidence-based decision-making, and global support is also needed to improve data quality [35]. Moreover, research, development, and application of innovative technologies, including mobile applications, techniques for vector control, diagnostic tools [36], and vaccines against malaria [37], all of these demand greater contribution from capable stakeholders to improve the effectiveness of global anti-malaria DAH. For donors like China with abundant experience in combating malaria and those who are ready to make greater contributions, all of these are selectable paths for future projects. Meantime, while giving full play to their strengths, all the donors should act in a coordinated way to avoid fragmentation or redundancy, and thus accelerate the process of malaria control and elimination.

## CONCLUSIONS

Overall, China has made considerable bilateral contributions to malaria prevention and control in SSA, and the considerations of recipient countries’ capacity for infectious diseases-related health service provision, as implied by the results, complied with the global appeal of health equity, albeit requiring further emphasis. The forms of China’s anti-malaria DAH remain to be diversified, and projects for capacity building require more resource allocation. With further efforts, global donors shall specify their own niches and consequently make the anti-malaria DAH system more harmonised, holistic, and effective.

## Additional material


Online Supplementary Document
