# Supplementary material for: China’s anti-malaria development assistance for health to sub-Saharan Africa and its influencing factors: A panel analysis
Source: J Glob Health. 2024 Dec 20;14:04250. doi: 10.7189/jogh.14.04250 (PMC11658716; doi:10.7189/jogh.14.04250)
Supplement: Online Supplementary Document [file jogh-14-04250-s001.pdf]

Table S1 Affiliations of interviewees

Code of the interviewee	Affiliation
01	Chinese Academy of International Trade and Economic Cooperation
02	Chinese Academy of International Trade and Economic Cooperation
03	China Center for Disease Control and Prevention
04	China Center for Disease Control and Prevention
05	China Center for Disease Control and Prevention
06	China Center for Disease Control and Prevention
07	Jiangsu Provincial Center for Disease Control and Prevention
08	Guangzhou University of Chinese Medicine

Table S2 Univariable panel analysis of influencing factors of the total amount allocation

Category	Variable	Two-year lagged		Three-year lagged		Four-year lagged		Five-year lagged		Six-year lagged	
		Coefficient (95%CI)	*P*	Coefficient (95%CI)	*P*	Coefficient (95%CI)	*P*	Coefficient (95%CI)	*P*	Coefficient (95%CI)	*P*
Health and health system	DALY in number	0.009 (-0.067, 0.087)	0.810	0.008 (-0.071, 0.087)	0.839	0.005 (-0.078, 0.087)	0.914	0.000 (-0.086, 0.087)	0.994	-0.005 (-0.095, 0.085)	0.907
	Death in number	0.014 (-0.062, 0.090)	0.722	0.013 (-0.067, 0.092)	0.750	0.010 (-0.073, 0.093)	0.815	0.006 (-0.081, 0.092)	0.899	0.000 (-0.091, 0.090)	0.997
	Incidence in number	0.001 (-0.075, 0.077)	0.979	0.000 (-0.080, 0.080)	0.966	-0.001 (-0.085, 0.084)	0.986	-0.002 (-0.091, 0.087)	0.962	-0.005 (-0.099, 0.089)	0.921
	Prevalence in number	0.006 (-0.070, 0.082)	0.880	0.009 (-0.071, 0.088)	0.832	0.010 (-0.074, 0.093)	0.823	0.008 (-0.079, 0.096)	0.849	0.005 (-0.087, 0.097)	0.916
	DALY rate	0.063 (-0.013, 0.140)	0.105	**0.080 (0.000, 0.159)**	**0.050**	**0.084 (0.001, 0.167)**	**0.047**	**0.090* (0.003, 0.176)**	**0.043**	**0.090 (-0.001, 0.181)**	**0.053**
	Death rate	**0.088 (0.110, 0.165)**	**0.025**	**0.101 (0.021, 0.181)**	**0.013**	**0.102 (0.019, 0.185)**	**0.017**	**0.109* (0.022, 0.196)**	**0.014**	**0.106 (0.014, 0.198)**	**0.023**
	Incidence rate	0.028 (-0.049, 0.104)	0.476	0.042 (-0.038, 0.121)	0.307	0.061 (-0.023, 0.145)	0.155	**0.077 (-0.012, 0.165)**	**0.089**	**0.093* (0.000, 0.186)**	**0.050**
	Prevalence rate	0.059 (-0.017, 0.135)	0.131	**0.080 (0.000, 0.160)**	**0.050**	**0.098 (0.014, 0.182)**	**0.022**	**0.110* (0.022, 0.198)**	**0.014**	**0.115 (0.023, 0.207)**	**0.015**
	GGHE-D%CHE	-0.035 (-0.113, 0.042)	0.364	-0.020 (-0.101, 0.061)	0.631	-0.036 (-0.122, 0.050)	0.413	0.004 (-0.088, 0.096)	0.936	-0.008 (-0.106, 0.089)	0.866
	Infectious% GGHE-D	-0.041 (-0.118, 0.035)	0.290	-0.040 (-0.120, 0.041)	0.335	-0.019 (-0.104, 0.067)	0.667	-0.027 (-0.118, 0.064)	0.559	-0.029 (-0.126, 0.067)	0.555
	malaria% GGHE-D	0.041 (-0.036, 0.117)	0.298	0.061 (-0.019, 0.143)	0.138	0.042 (-0.044, 0.128)	0.339	0.073 (-0.020, 0.165)	0.122	**0.096 (-0.002, 0.194)**	**0.055**
	UHC-SCI	**-0.079 (-0.159, 0.000)**	**0.049**	**-0.112 (-0.197, -0.027)**	**0.009**	**-0.149 (-0.240, -0.058)**	**0.001**	**-0.172* (-0.271, -0.072)**	**0.001**	**-0.224 (-0.331, -0.117)**	**0.000**

	UHC sub-index for infectious diseases	**-0.097<br>(-0.178, -0.016)**	**0.019**	**-0.143<br>(-0.231, -0.056)**	**0.001**	**-0.208<br>(-0.302, -0.114)**	**0.000**	**-0.227*<br>(-0.332, -0.121)**	**0.000**	**-0.277<br>(-0.392, -0.162)**	**0.000**
Ecomonics and politics	Natural resource rent % GDP	0.009<br>(-0.066, 0.085)	0.804	0.026<br>(-0.053, 0.104)	0.522	0.009<br>(-0.073, 0.092)	0.827	-0.008<br>(-0.094, 0.077)	0.848	-0.017<br>(-0.106, 0.073)	0.716
	GNI per capita	-0.029<br>(-0.104, 0.047)	0.455	-0.048<br>(-0.127, 0.030)	0.226	**-0.074<br>(-0.155, 0.008)**	**0.078**	**-0.091<br>(-0.177, -0.006)**	**0.037**	**-0.101*<br>(-0.191, -0.010)**	**0.029**
	TAC in the public sector rating	**0.080<br>(0.002, 0.157)**	**0.044**	**0.079<br>(-0.003, 0.161)**	**0.059**	**0.092*<br>(0.005, 0.178)**	**0.038**	**0.095*<br>(0.003, 0.187)**	**0.043**	**0.090<br>(-0.008, 0.188)**	**0.072**
	PSA: Estimate	0.018<br>(-0.058, 0.094)	0.647	0.018<br>(-0.061, 0.098)	0.651	0.006<br>(-0.078, 0.089)	0.890	0.023<br>(-0.065, 0.111)	0.612	0.013<br>(-0.079, 0.106)	0.778
Relationship with China	China's exports	-0.030<br>(-0.115, 0.054)	0.478	-0.051<br>(-0.142, 0.040)	0.270	-0.078<br>(-0.176, 0.019)	0.116	**-0.100<br>(-0.205, 0.006)**	**0.064**	**-0.109*<br>(-0.222, 0.005)**	**0.060**
	China's imports	-0.029<br>(-0.105, 0.046)	0.446	-0.037<br>(-0.116, 0.043)	0.369	-0.046<br>(-0.131, 0.039)	0.290	-0.056<br>(-0.146, 0.034)	0.225	-0.064<br>(-0.158, 0.030)	0.179
	China's investment	-0.056<br>(-0.144, 0.032)	0.215	**-0.101<br>(-0.200, -0.001)**	**0.048**	**-0.118<br>(-0.237, 0.001)**	**0.053**	**-0.145<br>(-0.285, -0.005)**	**0.042**	**-0.186*<br>(-0.340, -0.031)**	**0.019**
	Number of Chinese workers	**-0.076<br>(-0.149, -0.002)**	**0.044**	**-0.080<br>(-0.157, -0.004)**	**0.040**	**-0.087*<br>(-0.168, -0.006)**	**0.036**	**-0.089<br>(-0.175, -0.003)**	**0.042**	**-0.090<br>(-0.181, 0.002)**	**0.054**
	Voting consistency in the UNGA	-0.038<br>(-0.114, 0.037)	0.321	-0.014<br>(-0.096, 0.067)	0.733	-0.043<br>(-0.129, 0.043)	0.326	-0.050<br>(-0.141, 0.041)	0.280	0.028<br>(-0.068, 0.124)	0.568
	Bi-directional diplomatic visits	0.010<br>(-0.064, 0.084)	0.798	-0.006<br>(-0.085, 0.074)	0.890	0.074<br>(-0.018, 0.167)	0.116	-0.021<br>(-0.118, 0.075)	0.664	-0.085<br>(-0.191, 0.022)	0.118

Note: * marks the model with the largest adjusted R^2 comparingly.

Table S3 Univariable panel analysis of influencing factors of medicine allocation

Category	Variable	Two-year lagged		Three-year lagged		Four-year lagged		Five-year lagged		Six-year lagged	
		Coefficient (95%CI)	*P*	Coefficient (95%CI)	*P*	Coefficient (95%CI)	*P*	Coefficient (95%CI)	*P*	Coefficient (95%CI)	*P*
Health and health system	DALY in number	0.001 (-0.076, 0.077)	0.985	0.001 (-0.078, 0.080)	0.981	0.000 (-0.082, 0.082)	0.998	-0.002 (-0.088, 0.084)	0.966	-0.003 (-0.092, 0.097)	0.953s
	Death in number	0.003 (-0.073, 0.080)	0.935	0.003 (-0.077, 0.082)	0.945	0.002 (-0.081, 0.085)	0.965	0.000 (-0.086, 0.086)	0.998	-0.002 (-0.092, 0.089)	0.972
	Incidence in number	-0.006 (-0.093, 0.070)	0.874	-0.008 (-0.088, 0.073)	0.850	-0.010 (-0.094, 0.075)	0.821	-0.011 (-0.100, 0.078)	0.804	-0.014 (-0.107, 0.080)	0.775
	Prevalence in number	0.002 (-0.074, 0.078)	0.967	0.002 (-0.078, 0.081)	0.959	0.001 (-0.082, 0.084)	0.978	0.000 (-0.087, 0.087)	0.996	-0.002 (-0.094, 0.088)	0.949
	DALY rate	0.021 (-0.055, 0.098)	0.580	0.038 (-0.040, 0.118)	0.338	0.052 (-0.030, 0.135)	0.216	0.055 (-0.031, 0.141)	0.211	0.067 (-0.023, 0.157)	0.147
	Death rate	0.034 (-0.042, 0.111)	0.382	0.051 (-0.028, 0.131)	0.204	0.060 (-0.023, 0.143)	0.156	0.061 (-0.025, 0.148)	0.167	0.075 (-0.015, 0.166)	0.106
	Incidence rate	-0.002 (-0.079, 0.073)	0.941	0.008 (-0.071, 0.088)	0.827	0.027 (-0.056, 0.111)	0.525	0.037 (-0.051, 0.125)	0.410	0.043 (-0.049, 0.136)	0.357
	Prevalence rate	0.026 (-0.050, 0.102)	0.505	0.038 (-0.041, 0.118)	0.348	0.052 (-0.031, 0.136)	0.218	0.063 (-0.024, 0.151)	0.154	0.072 (-0.020, 0.164)	0.125
	GGHE-D%CHE	**-0.076 (-0.153, 0.001)**	**0.054**	-0.068 (-0.149, 0.013)	0.101	**-0.076* (-0.161, 0.009)**	**0.082**	-0.063 (-0.154, 0.027)	0.173	-0.063 (-0.161, 0.033)	0.197
	Infectious% GGHE-D	-0.001 (-0.078, 0.075)	0.972	0.009 (-0.071, 0.090)	0.819	0.044 (-0.040, 0.130)	0.304	0.033 (-0.056, 0.123)	0.467	0.025 (-0.071, 0.121)	0.609
	malaria% GGHE-D	0.030 (-0.046, 0.107)	0.440	0.065 (-0.015, 0.147)	0.111	0.067 (-0.018, 0.153)	0.124	0.066 (-0.026, 0.158)	0.160	**0.100 (0.002, 0.198)**	**0.043**
	UHC-SCI	-0.065 (-0.144, 0.013)	0.106	**-0.124 (-0.209, -0.039)**	**0.004**	**-0.133 (-0.224, -0.042)**	**0.004**	**-0.165* (-0.264, -0.066)**	**0.001**	**-0.222 (-0.329, -0.115)**	**0.000**
	UHC sub-index for	-0.032 (-0.113, 0.049)	0.438	**-0.111 (-0.199, -0.023)**	**0.013**	**-0.126 (-0.221, -0.031)**	**0.009**	**-0.158* (-0.264, -0.052)**	**0.003**	**-0.210 (-0.326, -0.095)**	**0.000**

	infectious diseases										
Ecomonics and politics	Natural resource rent % GDP	0.024 (-0.050, 0.099)	0.521	0.071 (-0.006, 0.149)	0.074	0.053 (-0.028, 0.136)	0.199	0.025 (-0.060, 0.110)	0.562	-0.001 (-0.090, 0.087)	0.973
	GNI per capita	-0.045 (-0.139, 0.017)	0.126	-0.061 (-0.139, 0.017)	0.126	**-0.079 (-0.161, 0.002)**	**0.056**	**-0.096 (-0.181, -0.010)**	**0.027**	**-0.103* (-0.193, -0.013)**	**0.024**
	TAC in the public sector rating	0.000 (-0.077, 0.078)	0.994	-0.016 (-0.098, 0.065)	0.691	0.025 (-0.061, 0.112)	0.566	-0.002 (-0.094, 0.089)	0.955	-0.008 (-0.106, 0.088)	0.862
	PSA: Estimate	-0.057 (-0.133, 0.018)	0.136	-0.057 (-0.136, 0.022)	0.158	-0.069 (-0.152, 0.013)	0.101	-0.048 (-0.136, 0.038)	0.276	-0.057 (-0.150, 0.034)	0.218
Relationship with China	China's exports	-0.034 (-0.118, 0.049)	0.426	-0.049 (-0.141, 0.041)	0.284	-0.079 (-0.177, 0.018)	0.110	**-0.103 (-0.208, 0.001)**	**0.054**	**-0.118* (-0.231, -0.005)**	**0.040**
	China's imports	-0.023 (-0.099, 0.052)	0.543	-0.027 (-0.106, 0.052)	0.506	-0.038 (-0.123, 0.046)	0.374	-0.053 (-0.143, 0.036)	0.241	-0.070 (-0.163, 0.023)	0.140
	China's investment	-0.066 (-0.154, 0.021)	0.140	**-0.095 (-0.195, 0.003)**	**0.059**	-0.078 (-0.197, 0.040)	0.197	**-0.148* (-0.287, -0.008)**	**0.037**	**-0.201 (-0.355, -0.047)**	**0.011**
	Number of Chinese workers	-0.056 (-0.129, 0.017)	0.135	**-0.070 (-0.147, 0.005)**	**0.071**	**-0.088 (-0.169, -0.007)**	**0.033**	**-0.094* (-0.180, -0.009)**	**0.030**	**-0.094 (-0.186, -0.003)**	**0.041**
	Voting consistency in the UNGA	0.002 (-0.073, 0.078)	0.944	-0.017 (-0.098, 0.064)	0.674	0.031 (-0.053, 0.117)	0.469	-0.019 (-0.109, 0.071)	0.681	0.065 (-0.030, 0.160)	0.183
	Bi-directional diplomatic visits	-0.022 (-0.097, 0.051)	0.543	0.061 (-0.018, 0.140)	0.132	-0.016 (-0.109, 0.076)	0.728	-0.076 (-0.172, 0.019)	0.118	-0.017 (-0.123, 0.088)	0.741

Note: * marks the model with the largest adjusted R^2 comparingly.

Table S4 Univariable panel analysis of influencing factors of the total amount allocation, with missing values dropped

Category	Variable	Two-year lagged		Three-year lagged		Four-year lagged		Five-year lagged		Six-year lagged	
		Coefficient (95%CI)	*P*	Coefficient (95%CI)	*P*	Coefficient (95%CI)	*P*	Coefficient (95%CI)	*P*	Coefficient (95%CI)	*P*
Health and health system	DALY in number	-0.023 (-0.274, 0.226)	0.985	-0.036 (-0.287, 0.214)	0.981	-0.051 (-0.303, 0.200)	0.998	-0.002 (-0.088, 0.084)	0.966	-0.003 (-0.092, 0.097)	0.953
	Death in number	0.003 (-0.255, 0.262)	0.935	-0.007 (-0.265, 0.250)	0.945	0.002 (-0.081, 0.085)	0.965	0.000 (-0.086, 0.086)	0.998	-0.002 (-0.092, 0.089)	0.972
	Incidence in number	-0.039 (-0.093, 0.070)	0.874	-0.042 (-0.300, 0.216)	0.850	-0.010 (-0.094, 0.075)	0.821	-0.011 (-0.100, 0.078)	0.804	-0.014 (-0.107, 0.080)	0.775
	Prevalence in number	00.002 (-0.297, 0.217)	0.967	-0.035 (-0.271, 0.200)	0.959	0.001 (-0.082, 0.084)	0.978	0.000 (-0.087, 0.087)	0.996	-0.002 (-0.094, 0.088)	0.949
	DALY rate	-0.037 (-00.055, 00.098)	0.580	0.129 (-0.155, 0.415)	0.338	0.052 (-0.030, 0.135)	0.216	0.055 (-0.031, 0.141)	0.211	0.067 (-0.023, 0.157)	0.147
	Death rate	0.134 (-0.153, 0.423)	0.382	0.143 (-0.119, 0.406)	0.204	0.060 (-0.023, 0.143)	0.156	0.061 (-0.025, 0.148)	0.167	0.075 (-0.015, 0.166)	0.106
	Incidence rate	0.154 (-0.109, 0.418)	0.941	-0.042 (-0.300, 0.216)	0.827	0.027 (-0.056, 0.111)	0.525	0.037 (-0.051, 0.125)	0.410	0.043 (-0.049, 0.136)	0.357
	Prevalence rate	00.026 (-00.050, 00.102)	0.505	-0.035 (-0.271, 0.200)	0.348	0.052 (-0.031, 0.136)	0.218	0.063 (-0.024, 0.151)	0.154	0.072 (-0.020, 0.164)	0.125
	GGHE-D%CHE	0.068 (-0.203, 0.340)	0.054	0.103 (-0.167, 0.374)	0.101	-0.076 (-0.161, 0.009)	0.612	-0.063 (-0.154, 0.027)	0.173	-0.063 (-0.161, 0.033)	0.197
	Infectious% GGHE-D	**-0.227 (-0.487, 0.033)**	**0.087**	**-0.256 (-0.515, 0.001)**	**0.052**	-0.215 (-0.481, 0.051)	0.304	**-0.231 (-0.495, 0. 032)**	**0.085**	**-0.227 (-0.494, 0.039)**	**0.094**
	malaria% GGHE-D	0.065 (-0.189, 0.320)	0.612	0.103 (-0.157, 0.365)	0.111	0.067 (-0.018, 0.153)	0.124	0.066 (-0.026, 0.158)	0.160	0.100 (-0.502, 0.198)	0.243
	UHC-SCI	-0.161 (-0.491, 0.169)	0.106	-0.126 (-0.458, -0.205)	0.542	-0.187 (-0.525, 0.149)	0.274	-0.165 (-0.264, 0.189)	0.383	-0.222 (-0.329, 0.132)	0.216
	UHC sub-index for	-0.262 (-0.601, 0.076)	0.438	**-0.306 (-0.633, 0.019)**	**0.065**	**-0.447 (-0.787, -0.106)**	**0.010**	**-.321 (-.671, .029)**	**0.072**	**-0.327 (-0.696, 0.040)**	**0.081**

	infectious diseases										
Ecomonics and politics	Natural resource rent % GDP	-0.042 (-0.272, 0.188)	0.521	-0.012 (-0.235, 0.210)	0.914	-0.038 (-0.274, 0.198)	0.199	0.025 (-0.060, 0.110)	0.562	-0.001 (-0.090, 0.087)	0.973
	GNI per capita	-0.045 (-0.293, 0.202)	0.126	-0.070 (-0.358, 0.216)	0.126	-0.144 (-0.506, 0.217)	0.433	-0.096 (-0.181, 0.238)	0.382	-0.103 (-0.253, 0.04)	0.224
	TAC in the public sector rating	**0.259 (0.013, 0.506)**	**0.039**	**0.303 (0.047, 0.558)**	**0.020**	**0.349 (0.090, 0.608)**	**0.008**	-0.002 (-0.094, 0.089)	0.955	**0.303 (0.045, 0.560)**	**0.021**
	PSA: Estimate	0.149 (-0.092, 0.390)	0.136	0.144 (-0.092, 0.382)	0.158	0.155 (-0.088, 0.399)	0.101	-0.048 (-0.136, 0.038)	0.276	-0.057 (-0.150, 0.034)	0.218
Relationship with China	China’s exports	-0.016 (-0.459, 0.426)	0.426	-0.100 (-0.539, 0.338)	0.284	-0.201 (-0.780, 0.377)	0.494	-0.103 (-0.208, 0.001)	0.154	-0.218 (-0.531, 0.005)	0.449
	China’s imports	-0.130 (-0.432, 0.170)	0.543	-0.124 (-0.382, 0.133)	0.506	-0.141 (-0.437, 0.154)	0.374	-0.053 (-0.143, 0.036)	0.241	-0.070 (-0.163, 0.023)	0.140
	China’s investment	-0.135 (-0.593, 0.323)	0.140	-0.193 (-0.672, 0.286)	0.428	-0.361 (-0.964, 0.241)	0.197	-0.148 (-0.287, 0.008)	0.237	-0.729 (-1.722, 0.264)	0.149
	Number of Chinese workers	-0.197 (-0.496, 0.101)	0.135	-0.167 (-0.491, 0.156)	0.309	-0.173 (-0.501, 0.155)	0.300	-0.094 (-0.180, 0.009)	0.140	-0.172 (-0.587, 0.242)	0.413
	Voting consistency in the UNGA	00.002 (-00.073, 00.078)	0.944	-0.119 (-0.365, 0.126)	0.674	-0.198 (-0.439, 0.042)	0.469	-0.019 (-0.109, 0.071)	0.681	0.065 (-0.030, 0.160)	0.183
	Bi-directional diplomatic visits	-00.022 (-00.097, 00.051)	0.543	-0.029 (-0.272, 0.213)	0.810	0.112 (-0.148, 0.372)	0.398	-0.076 (-0.172, 0.019)	0.118	-0.017 (-0.123, 0.088)	0.741

Table S5 Univariable panel analysis of influencing factors of medicine allocation, with missing values dropped

Category	Variable	Two-year lagged		Three-year lagged		Four-year lagged		Five-year lagged		Six-year lagged	
		Coefficient (95%CI)	*P*	Coefficient (95%CI)	*P*	Coefficient (95%CI)	*P*	Coefficient (95%CI)	*P*	Coefficient (95%CI)	*P*
Health and health system	DALY in number	0.009 (-0.067, 0.087)	0.810	0.008 (-0.071, 0.087)	0.839	0.005 (-0.078, 0.087)	0.914	0.000 (-0.086, 0.087)	0.994	-0.005 (-0.095, 0.085)	0.907
	Death in number	0.014 (-0.062, 0.090)	0.722	0.013 (-0.067, 0.092)	0.750	0.010 (-0.073, 0.093)	0.815	0.006 (-0.081, 0.092)	0.899	0.000 (-0.091, 0.090)	0.997
	Incidence in number	0.001 (-0.075, 0.077)	0.979	0.000 (-0.080, 0.080)	0.966	-0.001 (-0.085, 0.084)	0.986	-0.002 (-0.091, 0.087)	0.962	-0.005 (-0.099, 0.089)	0.921
	Prevalence in number	0.006 (-0.070, 0.082)	0.880	0.009 (-0.071, 0.088)	0.832	0.010 (-0.074, 0.093)	0.823	0.008 (-0.079, 0.096)	0.849	0.005 (-0.087, 0.097)	0.916
	DALY rate	0.063 (-0.013, 0.140)	0.105	0.080 (0.000, 0.159)	0.050	-0.241 (-0.568, 0.086)	0.147	-0.264 (-0.588, 0.059)	0.109	-0.255 (-0.586, 0.075)	0.129
	Death rate	0.088 (-0.110, 0.165)	0.305	0.101 (0.021, 0.181)	0.013	-0.192 (-0.496, 0.110)	0.212	-0.223 (0.022, 0.196)	0.014	-0.152 (-0.461, 0.157)	0.333
	Incidence rate	0.028 (-0.049, 0.104)	0.476	0.042 (-0.038, 0.121)	0.307	0.061 (-0.023, 0.145)	0.155	0.077 (-0.526, 0.078)	0.146	-0.208 (-0.516, 0.099)	0.183
	Prevalence rate	0.059 (-0.017, 0.135)	0.131	-0.118 (-0.375, 0.139)	0.366	-0.124 (-0.385, 0.135)	0.346	-0.132 (-0.399, 0.134)	0.328	-0.141 (-0.418, 0.134)	0.313
	GGHE-D%CHE	-0.035 (-0.113, 0.042)	0.364	**-0.040 (-0.101, 0.002)**	**0.061**	-0.036 (-0.122, 0.050)	0.413	0.004 (-0.088, 0.096)	0.936	-0.008 (-0.106, 0.089)	0.866
	Infectious% GGHE-D	-0.041 (-0.118, 0.035)	0.290	-0.040 (-0.120, 0.041)	0.335	-0.019 (-0.104, 0.067)	0.667	-0.027 (-0.118, 0.064)	0.559	-0.029 (-0.126, 0.067)	0.555
	malaria% GGHE-D	0.041 (-0.036, 0.117)	0.298	0.061 (-0.019, 0.143)	0.138	0.042 (-0.044, 0.128)	0.339	0.073 (-0.020, 0.165)	0.122	-0.036 (-0.345, 0.271)	0.814
	UHC-SCI	-0.079 (-0.369, 0.211)	0.290	-0.112 (-0.197, 0.027)	0.142	0.122 (-0.283, 0.529)	0.551	0.113 (-0.257, 0.364)	0.462	-0.164 (-0.573, 0.101)	0.672
	UHC sub-index for infectious diseases	**-0.197 (-0.320, -0.448)**	**0.092**	**-0.287 (-0.505, 0.000)**	**0.051**	-0.015 (-0.288, 0.118)	0.573	**-0.232 (-0.303, -0.089)**	**0.035**	0.236 (-0.200, 0.674)	0.286

Ecomonics and politics	Natural resource rent % GDP	0.009 (-0.066, 0.085)	0.804	0.026 (-0.053, 0.104)	0.522	0.009 (-0.073, 0.092)	0.827	-0.008 (-0.094, 0.077)	0.848	-0.017 (-0.106, 0.073)	0.716
	GNI per capita	-0.029 (-0.104, 0.047)	0.455	-0.048 (-0.127, 0.030)	0.226	**-0.093 (-0.488, 0.010)**	**0.081**	-0.083 (-0.553, -0.388)	0.726	-0.051 (-0.506, 0.403)	0.822
	TAC in the public sector rating	0.010 (-0.251, 0.271)	0.940	0.036 (-0.241, 0.314)	0.796	0.030 (-0.254, 0.314)	0.835	0.180 (-0.108, 0.468)	0.219	0.090 (-0.228, 0.335)	0.702
	PSA: Estimate	0.018 (-0.058, 0.094)	0.647	0.018 (-0.061, 0.098)	0.651	0.006 (-0.078, 0.089)	0.890	0.023 (-0.065, 0.111)	0.612	0.013 (-0.079, 0.106)	0.778
Relationship with China	China's exports	-0.030 (-0.115, 0.054)	0.478	**-0.051 (-0.242, 0.004)**	**0.070**	**-0.078 (-0.176, 0.009)**	**0.096**	-0.423 (-0.805, 0.106)	0.664	-0.109 (-0.322, 0.095)	0.360
	China's imports	-0.029 (-0.105, 0.046)	0.446	-0.037 (-0.116, 0.043)	0.369	-0.046 (-0.131, 0.039)	0.290	-0.056 (-0.146, 0.034)	0.225	-0.064 (-0.158, 0.030)	0.179
	China's investment	-0.056 (-0.144, 0.032)	0.215	**-0.338 (-0.968, 0.092)**	**0.091**	**-0.118 (-0.437, 0.031)**	**0.053**	-0.068 (-0.585, 0.397)	0.442	-0.027 (-0.340, 0.231)	0.758
	Number of Chinese workers	0.076 (-0.259, 0.082)	0.244	-0.248 (-0.771, 0.275)	0.351	**-0.450 (-1.330, 0.029)**	**0.072**	-0.467 (-1.879, 0.944)	0.512	**-0.685 (-2.068, 0.697)**	**0.086**
	Voting consistency in the UNGA	-0.038 (-0.114, 0.037)	0.321	-0.014 (-0.096, 0.067)	0.733	-0.043 (-0.129, 0.043)	0.326	-0.050 (-0.141, 0.041)	0.280	0.028 (-0.068, 0.124)	0.568
	Bi-directional diplomatic visits	0.010 (-0.064, 0.084)	0.798	-0.006 (-0.085, 0.074)	0.890	0.074 (-0.018, 0.167)	0.116	-0.021 (-0.118, 0.075)	0.664	-0.085 (-0.191, 0.022)	0.118
